# TUBA4A downregulation as observed in ALS *post-mortem* motor cortex causes ALS-related abnormalities in zebrafish

**DOI:** 10.3389/fncel.2024.1340240

**Published:** 2024-02-21

**Authors:** Evelien Van Schoor, Dufie Strubbe, Elke Braems, Jochen Weishaupt, Albert C. Ludolph, Philip Van Damme, Dietmar Rudolf Thal, Valérie Bercier, Ludo Van Den Bosch

**Affiliations:** ^1^Laboratory of Neuropathology, Department of Imaging and Pathology, KU Leuven (University of Leuven) and Leuven Brain Institute (LBI), Leuven, Belgium; ^2^Laboratory of Neurobiology, Department of Neurosciences, KU Leuven (University of Leuven) and Leuven Brain Institute (LBI), Leuven, Belgium; ^3^Center for Brain and Disease Research, VIB, Leuven, Belgium; ^4^Department of Neurology, Ulm University, Ulm, Germany; ^5^Deutsches Zentrum für Neurodegenerative Erkrankungen, Ulm, Germany; ^6^Department of Neurology, University Hospitals Leuven, Leuven, Belgium; ^7^Department of Pathology, University Hospitals Leuven, Leuven, Belgium

**Keywords:** amyotrophic lateral sclerosis, TUBA4A, microtubules, axonal pathology, zebrafish

## Abstract

Disease-associated variants of *TUBA4A* (alpha-tubulin 4A) have recently been identified in familial ALS. Interestingly, a downregulation of TUBA4A protein expression was observed in familial as well as sporadic ALS brain tissue. To investigate whether a decreased TUBA4A expression could be a driving factor in ALS pathogenesis, we assessed whether *TUBA4A* knockdown in zebrafish could recapitulate an ALS-like phenotype. For this, we injected an antisense oligonucleotide morpholino in zebrafish embryos targeting the zebrafish *TUBA4A* orthologue. An antibody against synaptic vesicle 2 was used to visualize motor axons in the spinal cord, allowing the analysis of embryonic ventral root projections. Motor behavior was assessed using the touch-evoked escape response. In *post-mortem* ALS motor cortex, we observed reduced TUBA4A levels. The knockdown of the zebrafish *TUBA4A* orthologue induced a motor axonopathy and a significantly disturbed motor behavior. Both phenotypes were dose-dependent and could be rescued by the addition of human wild-type *TUBA4A* mRNA. Thus, *TUBA4A* downregulation as observed in ALS *post-mortem* motor cortex could be modeled in zebrafish and induced a motor axonopathy and motor behavior defects reflecting a motor neuron disease phenotype, as previously described in embryonic zebrafish models of ALS. The rescue with human wild-type *TUBA4A* mRNA suggests functional conservation and strengthens the causal relation between TUBA4A protein levels and phenotype severity. Furthermore, the loss of TUBA4A induces significant changes in post-translational modifications of tubulin, such as acetylation, detyrosination and polyglutamylation. Our data unveil an important role for *TUBA4A* in ALS pathogenesis, and extend the relevance of *TUBA4A* to the majority of ALS patients, in addition to cases bearing *TUBA4A* mutations.

## 1 Introduction

Amyotrophic lateral sclerosis (ALS) is a fatal neurodegenerative disorder characterized by progressive paralysis resulting from the selective loss of upper and lower motor neurons. Patients usually die 2–5 years after disease onset due to respiratory failure. ALS has an incidence of 1–2 individuals per 100.000 each year. About 90% of patients display sporadic ALS, with no family history of the disease. In the remaining 10%, the disease is transmitted within families, referred to as familial ALS (Taylor et al., 2016). The most common disease-causing mutations are found in fused in sarcoma (*FUS*), superoxide dismutase 1 (*SOD1*), chromosome 9 open reading frame 72 (*C9orf72*) and transactive response DNA-binding protein (*TARDBP*) (Al-Chalabi and Hardiman, 2013; Taylor et al., 2016).

In addition, several genes with a role in cytoskeletal dynamics and axonal transport are linked to ALS, amongst which dynactin subunit 1 (*DCTN1*), kinesin family member 5A (*KIF5A*) and spastin (*SPAST*) (Castellanos-Montiel et al., 2020). This suggests that there might be a direct causative relationship between defects in cytoskeletal integrity and neurodegeneration. More recently, Smith et al. found mutated variants in the alpha-tubulin 4A (*TUBA4A*) gene in ALS patients, based on exome sequencing data from a large cohort of ALS patients and controls. These mutated variants are associated with classical spinal onset ALS, and in some cases also frontotemporal dementia (FTD)-like symptoms (Smith et al., 2014). We confirmed the importance of *TUBA4A* variants in ALS in an independent Belgian cohort (Perrone et al., 2017).

*TUBA4A* encodes one of nine known α-tubulin isotypes, with all variants expressed from different genes. The structures of α- and β-tubulin are highly conserved throughout eukaryotes, nevertheless, the range of human diseases associated with mutations in different tubulin isotypes indicates that specific isotypes have different functional specifications (Breuss et al., 2017). This is supported by the fact that the expression of different isotypes differs depending on cell type and tissue. For example, *TUBA8A* is mainly expressed in testes and skeletal muscle, while *TUBA4A* is highly expressed in the nervous system (Braun et al., 2010; Clark et al., 2016).

Functionally, α-tubulin assembles with β-tubulin to form stable tubulin heterodimers, which dynamically polymerize into sheets of longitudinal polarized protofilaments, building the cylindrical, hollow microtubules (Tischfield et al., 2011; Chakraborti et al., 2016). Stable microtubules are important for a wide range of functions in long extending axons, and serve as the tracks along which motor proteins (such as dynein and kinesin) move cargoes with the help of adaptor proteins (Clark et al., 2016). Hereby, microtubule stability and function is influenced by the presence of different post-translational modifications (PTMs) of tubulin (Boiarska and Passarella, 2021). Most PTMs are reversible and occur on the C-terminal tail of tubulins, except for lysine (K40) acetylation which is located on the luminal surface or microtubules (Sferra et al., 2020).

Motor neurons are the most asymmetric cells in nature, with axons reaching a meter in length in humans. Therefore, they have a crucial requirement for proper cytoskeletal functioning. A disruption of cytoskeleton integrity could affect cell morphology, axonal branching, the establishment of neuromuscular junctions, and many other critical cell functions. In addition, it could prevent molecular motors from transporting the necessary cargoes, with a potentially deleterious effect on neuronal function (Clark et al., 2016; Taylor et al., 2016; Castellanos-Montiel et al., 2020). Interestingly, it was reported that sporadic ALS patients have a downregulation of α-tubulin subunits in affected brain regions (Jiang et al., 2005; Helferich et al., 2018; Maraldi et al., 2019). However, whether these alterations in α-tubulin expression in the majority of ALS patients can also drive ALS disease pathogenesis is still unknown.

In this study, we confirmed a decrease in TUBA4A protein expression in *post-mortem* tissue from ALS patients compared to controls. We mimicked this decrease in zebrafish using antisense oligonucleotide morpholinos (AMO) directed against *tuba8l2* (ENSDARG00000031164), the single zebrafish orthologue for *TUBA4A*, which is 94% conserved at the protein level. This decreased expression of tuba8l2 did not affect total levels of alpha-tubulin, but led to abnormalities in the axons of spinal cord motor neurons, as well as motor behavior deficits consistent with published models of ALS (Kabashi et al., 2011, Swinnen et al., 2018). Both phenotypes were dose-dependent and could be rescued by the addition of human wild-type *TUBA4A* mRNA. Additionally, while we did not observe changes in microtubule polymerization, we found significant changes in post-translational modifications of tubulin in our zebrafish knockdown model. Overall, our data point toward a central role of *TUBA4A* in ALS pathogenesis, aside from cases bearing *TUBA4A* mutations.

## 2 Material and Methods

### 2.1 Human autopsy cases

Brain and spinal cord tissues were collected in accordance with the applicable laws in Belgium (UZ Leuven) and Germany (Ulm). The recruitment protocols for collecting the brains were approved by the ethical committees of the University of Ulm (Germany) and UZ Leuven (Belgium). This study was approved by the UZ Leuven ethical committee (Belgium) (S60803, S55312). Tissues were collected with an average *post-mortem* interval of 45 h. After autopsy, the right hemisphere was dissected in coronal planes and frozen at −80^°^C. The left hemisphere was fixed in 4% phosphate-buffered formaldehyde (PFA) (F8775, Sigma-Aldrich, St Louis, MO, US). Ten sporadic ALS cases and twelve non-neurodegenerative controls were included in this study (Online resource Supplementary Table 1). The diagnosis of ALS was based on clinical assessment according to the consensus criteria for ALS (Brooks et al., 2000; de Carvalho et al., 2008; de Carvalho and Swash, 2009). The *post-mortem* diagnosis of ALS was pathologically confirmed by assessment of the pTDP-43 pathology. Braak NFT stage (Braak et al., 2006) and AβMTL phase (Thal et al., 2000) were determined based on immunohistochemical stainings with antibodies against Aβ and p-tau.

### 2.2 Human tissue immunohistochemistry

Histological examination was performed on 5 μm thick sections cut from formalin-fixed, paraffin-embedded tissue of frontal, pre- and post-central, and temporal cortex, hippocampus and spinal cord. Sections were stained with antibodies against pTDP-43, TUBA4A (C-term), pTau^(S202/T205)^ and Aβ_17–24_ (Online resource Supplementary Table 2). Stainings were performed with the BOND-MAX automated IHC/ISH Stainer (Leica Biosystems, Wetzlar, Germany) using the Bond Polymer Refine Detection kit (DS9800, Leica Biosystems). Briefly, slides were deparaffinized and epitopes were retrieved with low or high pH buffer. After incubation with Peroxidase-Blocking Reagent (DS9800, Leica Biosystems), slides were incubated with primary antibodies for 30 min, followed by secondary antibody incubation. DAB was used for visualization, followed by counterstaining with hematoxylin. Dehydration was carried out in an autostainer, followed by mounting in an automated cover-slipper (Leica Biosystems). Images were acquired using the Leica DM2000 LED microscope coupled to a Leica DFC 7000 T camera. Images were processed using ImageJ and combined into figures using Inkscape.

### 2.3 Human tissue protein extraction

For biochemistry of human tissues, the right hemispheres were cut in approx. 1 cm thick slabs and frozen at −80^°^C. Fifty mg of motor cortex and spinal cord was weighed and mechanically homogenized in 0.5 ml 2% SDS in TBS (Tris-buffered saline) with Nuclease (88701, Pierce™ Universal Nuclease, Thermo Fisher Scientific) and a cocktail of protease/phosphatase inhibitors (78440, Halt, Thermo Fisher Scientific) using a micropestle (CXH7.1, Carl Roth, Karlsruhe, Germany). Samples were sonicated, followed by a centrifugation at 13,000 g for 30 min. The resulting supernatant was used. Protein concentrations were determined using the Pierce BCA Protein Assay Kit (23225, Thermo Fisher Scientific).

### 2.4 Zebrafish protein extraction

Zebrafish embryos were collected at 48 h post fertilization (hpf) and were manually dechorionated using forceps and the yolk was removed. The embryos were homogenized in RIPA buffer (R0278, Sigma-Aldrich) supplemented with protease and phosphatase inhibitors (78440, Halt, Thermo Fisher Scientific) using a micropestle on a rotor. After centrifugation (3 min, 13 000 g), the supernatant was collected and protein concentrations were determined using the Pierce BCA Protein Assay Kit (23225, Thermo Fisher Scientific).

### 2.5 Human tissue and zebrafish western blotting

For western blotting, 10 μg (human central nervous system lysates) or 20 μg (zebrafish lysates) of protein was loaded on a Bis-Tris 4–12% gradient SDS-PAGE (WG1402BOX, Invitrogen, Thermo Fisher Scientific) in MOPS-SDS running buffer (J62847.K2, Alfa Aesar, Haverhill, MA, USA), electrophoresed at 150 V for 60 min, and transferred to a nitrocellulose membrane (GE10600001, Semidry transfer, Biorad, Hercules, CA, USA). Membranes were blocked with 5% non-fat dried milk (A0830.1000, AppliChem, Darmstadt, Germany) in phosphate-buffered saline (PBS) 0.1% Tween-20 (PBST). Primary antibodies and the corresponding dilutions are listed in Supplementary Table 2 (Online resource). Secondary antibodies were goat anti-rabbit IgG-HRP or goat anti-mouse IgG-HRP (1:10 000, P044801-2 and P044701-2, polyclonal, Dako). Blots were developed with SuperSignal West Pico or Dura plus ECL reagent (34580 and 34075, Thermo Fisher Scientific). Digital images were acquired using the Amersham Imager 600 (GE Healthcare, Chicago, IL, USA). All blots were stripped (21063, Restore Western Blot Stripping Buffer, Thermo Fisher Scientific) of bound antibodies and reprobed with GAPDH to control for equal protein loading. Band intensities were measured using ImageJ and were normalized to GAPDH.

### 2.6 Antisense oligonucleotide morpholino design and *TUBA4A* mRNA transcription

An ATG blocking morpholino (AMO) against *tuba8l2*, the single human *TUBA4A* orthologue in Danio rerio (morpholino sequence 5’-TTGGAGTTGGATTTGTTTTTTGCCG-3’) was designed and generated by Gene Tools (Philomath, USA). The standard control AMO provided by Gene Tools was used as negative control (morpholino sequence 5’- CCTCTTACCTCAGTTACAATTTATA-3’). A human wild-type TUBA4A HA-tagged encoding plasmid was kindly provided by Dr. J. Landers (Smith et al., 2014). To produce mRNA, plasmids were linearized by restriction digestion, transcribed with mMESSAGE mMACHINE T7 kit (AM1344, Ambion, Huntingdon, UK) and the resulting mRNA purified with the MEGAclear Kit (AM1908, Ambion). The mRNA concentration was measured by spectrophotometry (Nanodrop, Thermo Fisher Scientific). mRNA quality and length were verified by RNA gel electrophoresis.

### 2.7 Zebrafish injections

All zebrafish breeding was approved by the Ethical Committee for Animal Experimentation of the KU Leuven (P125/2014). Zebrafish were reared and incubated at 28.5^°^C. All experiments were performed on embryos younger than five days post fertilization, implying that these experiments are in line with the principle of 3Rs as these are not regulated as animal studies. One- to two-cell stage zebrafish embryos from the AB strain were injected with the indicated amounts of morpholino and/or mRNA diluted in aqua ad iniectabilia (3521664, B. Braun, Melsungen, Germany) and supplemented with phenol red for verification of injection volume.

### 2.8 Zebrafish SV2 immunohistochemistry and analysis

At 30 hpf, embryos were manually dechorionated and deyolked, and fixed overnight at 4^°^C in 4% PFA in PBS. Fish were permeabilized with acetone for 1 h at −20^°^C, followed by blocking with 1% bovine serum albumin (BSA) (A7030, Sigma-Aldrich)/1% dimethyl sulfoxide (DMSO) (D2650, Sigma-Aldrich)/PBS for 1h at RT and immunostained with mouse anti-synaptic vesicle 2 (SV2) (1:200; online resource Supplementary Table 2) and secondary Alexa Fluor 555 anti-mouse antibody (1:500, A-31570, Thermo Fisher Scientific) as previously described (Swinnen et al., 2018). For axonal length analysis, 10–15 embryos per condition per experiment were analyzed using a Leica DM 3000 LED microscope and the tracking tool in Lucia software (version 4.60, Laboratory Imaging, resolution 2448 x 2048 pixels). Five predefined and consecutive ventral root projections (i.e. the 8^th^ up to the 12^th^ axon) were measured by a blinded observer. Each axon was measured starting from the beginning of the ventral root projection until the end of any observable staining. Data were normalized to the control condition. A total of 10–15 embryos were used per condition per experiment with three biological replicates, which has previously been shown to be adequate to measure an effect (Swinnen et al., 2018).

### 2.9 Zebrafish touch-evoked escape response (TEER)

Embryos were manually dechorionated at 30 hpf. 10–15 embryos were used per condition per experiment with three biological replicates, which has previously been shown to be adequate to measure an effect (Bercier et al., 2019). At 48 hpf, zebrafish embryos were individually placed in a 150 mm petri dish filled with 28.5^°^C embryo medium. After 30 s of habituation, an escape response was elicited by a light brush on the tail and recorded at 30 Hz with a Sony HDR-AS30V camera (resolution 1920 x 1080 pixels) until the end of the escape response (Kabashi et al., 2011). The videos were analyzed in ImageJ using the Manual Tracking plugin and the total distance, the maximal instant velocity and the average velocity were calculated by a blinded observer. Data were normalized to the control condition.

### 2.10 EB3 comet assay

Analysis of microtubule polymerization events was performed according to the previously described EB3 comet assay (Bercier et al., 2019), where EB3-GFP is expressed in single caudal primary (CaP) motor neurons by microinjection of the pUAS-EB3-GFP plasmid in the Tg(mnx1:GAL4) line (Zelenchuk and Brusés, 2011). Time-lapse imaging was performed at 48 hpf on live, agarose-embedded embryos with a spinning disk confocal microscope (Nikon NiE microscope, Yokogawa CSU-X spinning-disk module and Teledyne Photometrics Prime 95B camera, NIS-Elements software, Nikon Instruments Europe B.V.). Imaging was performed using a 60x LWD water-immersion lens (Nikon Fluor 60x/NA 1.00 WD 2.0) where an image was acquired every 500 ms for a total duration of 5 min. The average length of the imaged arbor segments did not differ between conditions. Kymograms were extracted from time-lapse series on linear segments of CaP distal arbors using the Kymograph Tool (Montpellier RIO Imaging, CNRS, France). Each pixel on the Y-axis represents one timepoint projected against neurite length on the X-axis. Kymogram analysis was performed to determine the duration and distance of single comets as well as the average speed of polymerization and the number of polymerization events (i.e., comet density).

### 2.11 Statistical analysis

Statistical analyses were performed using Graphpad Prism 9.0 software. Normality was assessed using the Shapiro-Wilk test. Variance homogeneity was assessed using the F-test (for two groups) or the Bartlett’s test (for more than two groups). A Mann-Whitney test or unpaired t-test was used to compare two groups. A one-way ANOVA or Kruskal-Wallis test followed by Dunn’s or Dunnett’s multiple comparisons was used to determine the significant difference between multiple groups. Data are presented as mean ± SD or median ± IQR. Significance levels are indicated as follows: *p < 0.05, **p < 0.01, ***p < 0.001, ****p < 00001.

## 3 Results

### 3.1 ALS *post-mortem* motor cortex shows decreased TUBA4A expression

**FIGURE 1:**
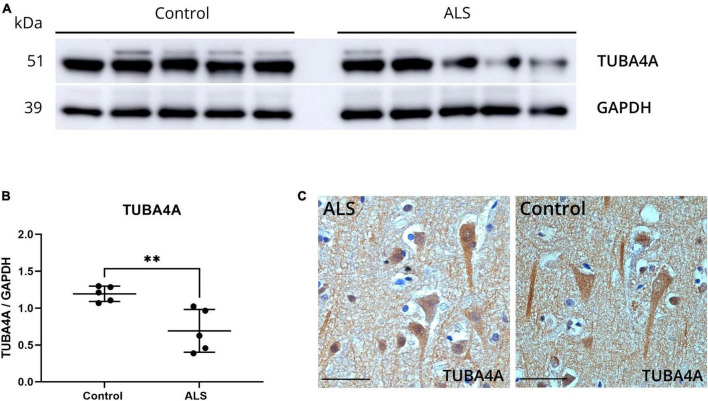
ALS *post-mortem* motor cortex exhibits TUBA4A downregulation. **(a)** Western blot on SDS-soluble lysates derived from the motor cortex of control (*n* = 5) and ALS (*n* = 5) cases using an antibody against TUBA4A (C-term). GAPDH was used as a loading control. **(b)** Quantification of the expression of TUBA4A as a ratio to GAPDH in the motor cortex. Unpaired t-test. **(c)** Immunohistochemical staining of the motor cortex of a representative ALS and control case with an antibody against TUBA4A (C-term). Scale bar represents 50 μm. **p < 0.01.

To investigate possible alterations in the expression of the TUBA4A protein in ALS, we performed western blot on SDS-soluble extracts from motor cortex from ALS and control cases using a TUBA4A-specific antibody. A significant decrease in the total protein expression of TUBA4A in ALS compared to control motor cortex was observed (*p* = 0.0066; unpaired t-test; Figures 1a, b; online resource Supplementary Figure 1a). In the spinal cord, there was a trend toward decreased TUBA4A levels in ALS cases compared to controls, although significance was not reached (*p* = 0.1349; unpaired t-test; online resource Supplementary Figures 1b, c). In addition, we evaluated the TUBA4A expression pattern by immunohistochemistry in ALS and control cases and observed a dense staining of the cell body and neurites in the motor cortex (Figure 1c) and in the spinal cord (online resource Supplementary Figure 2), both in ALS cases and controls. No TUBA4A inclusions were observed microscopically in the motor cortex or the spinal cord (Figure 1c, online resource Supplementary Figure 2). These results from *post-mortem* human tissue showed that TUBA4A protein levels were reduced in sporadic ALS patient tissue without changes in protein distribution.

### 3.2 Knockdown of zebrafish *TUBA4A* orthologue induces dose-dependent axonal abnormalities and motor behavior deficits in zebrafish

**FIGURE 2:**
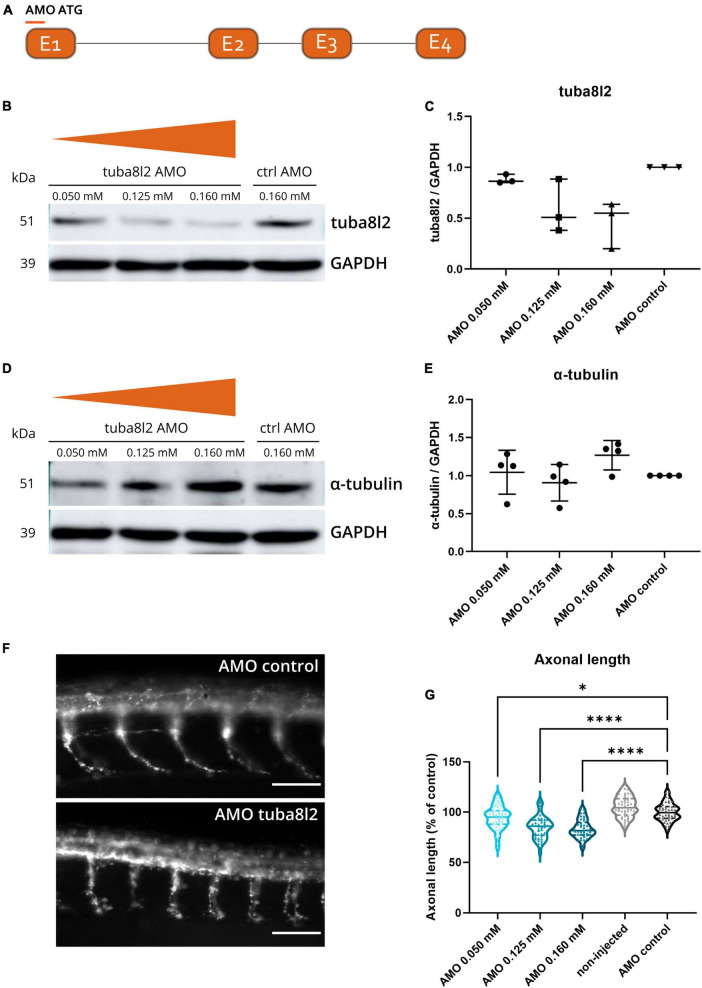
Specific *tuba8l2* knockdown in zebrafish induces axonal abnormalities. **(a)** An ATG morpholino was designed against the *Danio rerio TUBA4A* orthologue *tuba8l2*. **(b–e)** Western blot was performed at 48 hpf after injection of different doses of ATG morpholino against *tuba8l2* (0.160 mM, 0.125 mM and 0.050 mM) as well as the injection of a control morpholino (0.160 mM). *N* = 3 experiments; *n* = 10–15 zebrafish per group per experiment. Quantification of tuba8l2 panel **(c)** and α-tubulin panel **(e)** protein levels relative to GAPDH for the different injection conditions. **(f,g)** Visualization of motor axons by SV2 immunohistochemistry at 30 hpf after injection of different doses of ATG morpholino against *tuba8l2* (0.160 mM, 0.125 mM and 0.050 mM) or a control morpholino (0.160 mM). A non-injected condition was also included. Scale bar represents 50 μm *p* < 0.0001 (0.160 mM versus AMO control), *p* < 0.0001 (0.125 mM versus AMO control) and *p* < 0.0450 (0.050 mM versus AMO control); one-way ANOVA with Dunnett’s multiple comparisons. Axonal length was measured for *N* = 3 experiments; *n* = 10–15 zebrafish embryos per group per experiment; with every data point representing the average length of the five measured axons for each zebrafish embryo. *p < 0.05; ****p < 0.0001. AMO, morpholino; hpf, hours post fertilization.

To determine the potential significance of TUBA4A downregulation, we investigated whether the knockdown of *tuba8l2* is deleterious to motor axons of zebrafish embryos. This is the single zebrafish *TUBA4A* orthologue (ENSDARG00000031164), which is 94% conserved at the protein level when compared to human *TUBA4A*, and shown to be expressed throughout all anatomical structures in embryonic stages (at least until pec-fin stage at 72 hpf (Thisse and Thisse, 2004). We designed a morpholino directed against the ATG start codon of *tuba8l2* (Figure 2a) and injected different doses in one- to two-cell stage zebrafish oocytes. The highest dose of 0.160 mM was determined through a dose-response where we observed a high increase in morphological abnormalities together with a fast drop in survival at doses above 0.200 mM. The dose of 0.160 mM led to morphologically normal embryos (Figure 4a) and a standard control morpholino was injected at a dose equaling the highest dose of the *tuba8l2* morpholino (0.160 mM). We then assessed tuba8l2 protein levels by western blot at 48 hpf using a specific antibody against TUBA4A (C-term). We detected a dose-dependent knockdown of tuba8l2 levels, with the highest knockdown of 54% at 0.160 mM of morpholino (mean ratio to control: 0.46), a knockdown of 41% at a dose of 0.125 mM (mean ratio to control: 0.59) and a 12% knockdown when we injected 0.050 mM morpholino (mean ratio to control: 0.88) (Figures 2b, c; online resource Supplementary Figure 3). Importantly, the morpholino injection and subsequent reduction in tuba8l2 did not affect the levels of total α-tubulin, as shown by western blot with an antibody against α-tubulin (Figures 2d, e; online resource Supplementary Figure 3) suggesting compensation by other isotypes.

To assess the effect of the specific knockdown of *tuba8l2* on motor neuron axonal morphology, we performed SV2 immunohistochemistry to visualize the ventral roots projections of the spinal cord motor neurons at 30 hpf (Figure 2f) (Swinnen et al., 2018). We observed a significant reduction in axonal length in the 0.160 mM *tuba8l2* morpholino condition compared to the control morpholino condition (*p* < 0.0001; one-way ANOVA with Dunnett’s multiple comparisons; Figure 2g). This effect was dose-dependent, as shown by the 0.125 mM and 0.050 mM morpholino conditions (*p* < 0.0001 and *p* = 0.045, respectively; one-way ANOVA with Dunnett’s multiple comparisons; Figure 2g).

**FIGURE 3:**
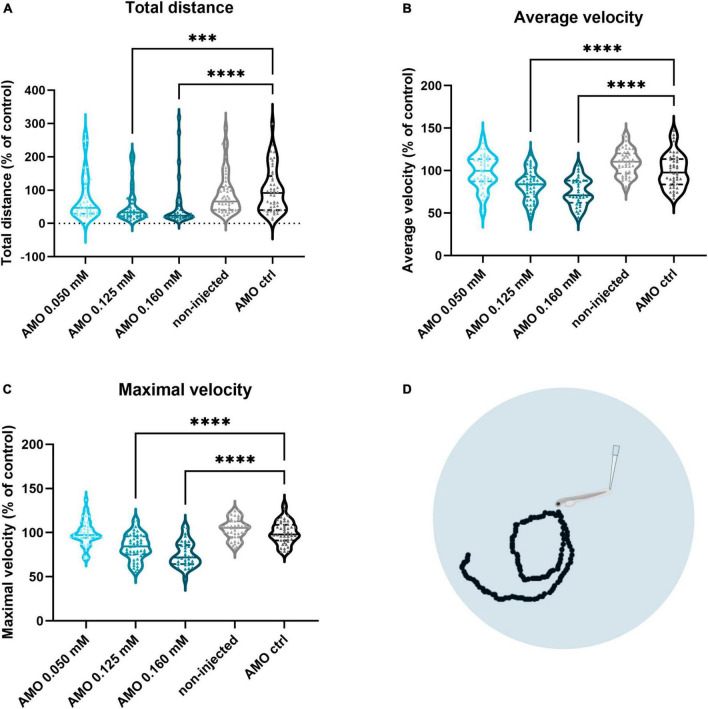
Zebrafish motor behavior deficits are induced by *tuba8l2* knockdown. Zebrafish were subjected to a touch-evoked escape response (TEER) assay at 48 hpf after injection of different doses of ATG morpholino against *tuba8l2* (0.160 mM, 0.125 mM, 0.050 mM), or a control morpholino (0.160 mM). In addition, non-injected embryos were included in the analysis. **(a)** Total distance for 0.160 mM (*p* < 0.0001), 0.125 mM (*p* = 0.0001) and 0.050 mM (*p* = 0.1936) compared to AMO control condition. **(b)** Average velocity for 0.160 mM (*p* < 0.0001), 0.125 mM (*p <* 0.0001) and 0.050 mM (*p* = 0.9814) compared to AMO control condition. **(c)** Maximal instant velocity 0.160 mM (*p* < 0.0001), 0.125 mM (*p* < 0.0001) and 0.050 mM (*p* = 0.9983) compared to AMO control condition. Kruskal-Wallis test with Dunn’s multiple comparisons panel **(a)** or one-way ANOVA with Dunnett’s multiple comparisons panels **(b,c)**; *N* = 3 experiments; *n* = 10–15 zebrafish embryos per group per experiment; with each data point representing an individual zebrafish embryo. **(d)** Visual example of the tracking of an escape response in the AMO control condition using the TEER assay in zebrafish embryos at 48 hpf. ***p < 0.001; ****p < 0.0001. AMO, morpholino; hpf, hours post fertilization.

To assess whether *tuba8l2* knockdown in zebrafish also had an effect on motor function, we performed a touch-evoked escape response (TEER) assay at 48 hpf as previously described (Kabashi et al., 2011). We compared non-injected, control morpholino injected and *tuba8l2* morpholino injected conditions, with an example escape trace of the AMO control condition depicted in Figure 3d. This assay showed that reduction of tuba8l2 led to a shorter escape, as shown by a significant decrease in total distance travelled (0.160 mM: *p* < 0.0001; 0.125 mM: *p* = 0.0001; Kruskal-Wallis test with Dunn’s multiple comparisons; Figure 3a). Furthermore, we observed a significant reduction in the average velocity (Figure 3b) and instant maximal velocity (Figure 3c) in the 0.160 mM (*p* < 0.0001; one-way ANOVA with Dunnett’s multiple comparisons) and the 0.125 mM (*p* < 0.0001; one-way ANOVA with Dunnett’s multiple comparisons) *tuba8l2* morpholino-injected embryos compared to control morpholino. In conclusion, we find that the specific knockdown of the zebrafish orthologue of *TUBA4A* led to a dose-dependent axonopathy and motor behavior phenotype similar to what has previously been described for zebrafish ALS models.

### 3.3 Axonal phenotype and motor behavior defects are rescued by human *TUBA4A* mRNA

**FIGURE 4:**
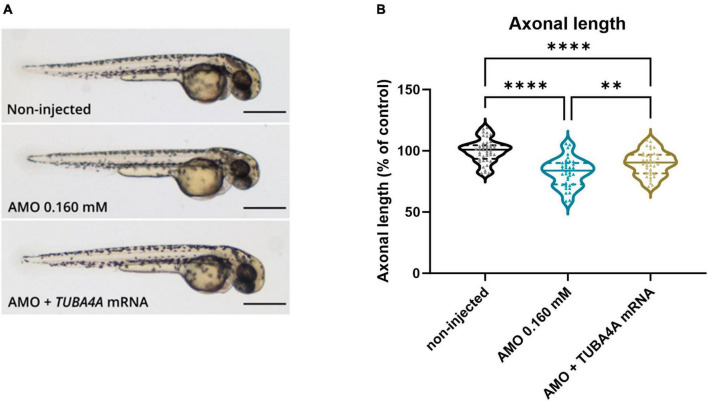
Rescue of axonal length defects by the addition of human wild-type *TUBA4A* mRNA. Zebrafish embryos were injected with an ATG morpholino against zebrafish *tuba8l2* (0.160 mM) with or without the injection of human wild-type *TUBA4A* mRNA (300 ng/μl). Non-injected embryos were also included in the analysis. **(a)** Representative whole body images of zebrafish embryos for the different conditions at 48 hpf (scale bar represents 500 μm). **(b)** At 30 hpf, axonal length was measured for all conditions, with *p* = 0.0051 (AMO 0.160 mM versus AMO 0.160 mM + *TUBA4A* mRNA), *p* < 0.0001 (AMO 0.160 mM versus non-injected) and *p* < 0.0001 (non-injected versus AMO 0.160 mM + *TUBA4A* mRNA); one-way ANOVA with Dunnett’s multiple comparisons. Axonal length was measured for N = 3 experiments; *n* = 10–15 zebrafish per group per experiment; with every data point representing the average length of the five measured axons for each zebrafish embryo. **p < 0.01; ****p < 0.0001. AMO, morpholino; hpf, hours post fertilization.

To confirm that the observed phenotypes are indeed a direct consequence of the specific knockdown of *tuba8l2*, and to confirm functional conservation between zebrafish and human orthologues, we assessed the rescue of these phenotypes through the co-expression of human wild-type TUBA4A. To achieve this, we injected zebrafish eggs with human HA-tagged *TUBA4A* mRNA at the highest non-toxic dose of 300 ng/μl, and collected the embryos for western blot at 48 hpf. An anti-HA antibody confirmed the expression of the HA-TUBA4A protein at 48 hpf in the *TUBA4A* mRNA-injected condition, which was absent in the control condition (Online resource; Supplementary Figure 4). Next, we co-injected *TUBA4A* mRNA with the highest *tuba8l2* morpholino dose (i.e.,0.160 mM) (Figure 4a). We analyzed the effect on spinal cord motor neurons, as aforementioned, by measuring axonal length using SV2 immunohistochemistry at 30 hpf. This showed a rescue of the phenotype by the co-injection of wild-type *TUBA4A* mRNA (Figure 4; *p* = 0.0051; one-way ANOVA with Dunnett’s multiple comparisons).

**FIGURE 5:**
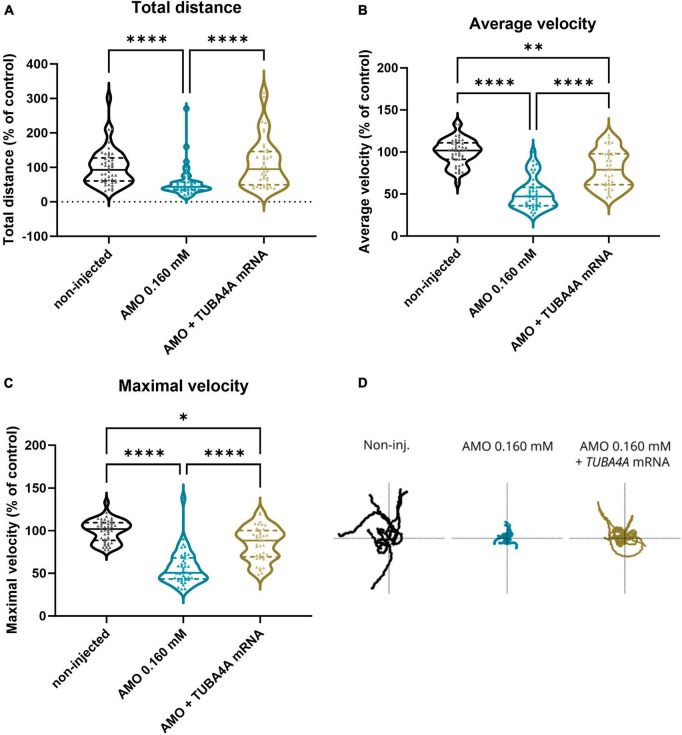
Rescue of motor behavior deficits by the addition of human wild-type *TUBA4A* mRNA. Zebrafish embryos were subjected to a touch-evoked escape response (TEER) assay at 48 hpf after injection with an ATG morpholino against zebrafish *tuba8l2* (0.160 mM) with or without the injection of wild-type human *TUBA4A* mRNA (300 ng/μl). Non-injected embryos were also included in the analysis. **(a)** Total distance for AMO 0.160 mM + *TUBA4A* mRNA compared to non-injected (non-significant) and AMO 0.160 mM condition (*p* < 0.0001), and non-injected (*p* < 0.0001) compared to AMO 0.160 mM condition. **(b)** Average velocity for AMO 0.160 mM + *TUBA4A* mRNA compared to non-injected (*p* = 0.0018) and AMO 0.160 mM condition (*p* < 0.0001), and non-injected (*p* < 0.0001) compared to AMO 0.160 mM condition. **(c)** Maximal instant velocity for AMO 0.160 mM + *TUBA4A* mRNA compared to non-injected (*p* = 0.0110) and AMO 0.160 mM condition (*p* < 0.0001), and non-injected (*p* < 0.0001) compared to AMO 0.160 mM condition. Kruskal-Wallis test with Dunn’s multiple comparisons; *N* = 3 experiments; *n* = 10–15 zebrafish embryos per group per experiment; which each data point representing an individual zebrafish embryo. **(d)** Representative escape traces from 10 zebrafish embryos per group shown as a visual example. *p < 0.05, **p < 0.01, and ****p < 0.0001. AMO, morpholino; hpf, hours post fertilization.

When we performed the TEER assay at 48 hpf, we also observed a complete rescue of the phenotype when looking at the total distance travelled (Figure 5a; *p* < 0.0001; Kruskal-Wallis test with Dunn’s multiple comparisons), and a partial rescue of the phenotype for the average swimming velocity (Figure 5b; *p* < 0.0001; Kruskal-Wallis test with Dunn’s multiple comparisons) and the instant maximal swimming velocity (Figure 5c; *p* < 0.0001; Kruskal-Wallis test with Dunn’s multiple comparisons). Representative escape traces for 10 embryos per group are depicted in Figure 5d. We showed that the observed axonopathy and behavioral phenotype induced by the knockdown of *tuba8l2* could be rescued by co-expression of human *TUBA4A* mRNA, confirming both the conservation between orthologues and the specificity of our knockdown approach.

### 3.4 Knockdown of zebrafish *tuba8l2* does not alter microtubule polymerization

**FIGURE 6:**
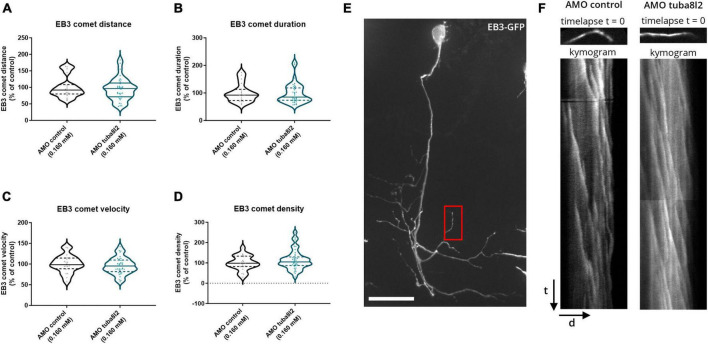
Zebrafish microtubule polymerization is not affected by *tuba8l2* knockdown. At 48 hpf, an EB3 comet assay was performed in single CaP motor neurons after co-injection of an ATG morpholino against *tuba8l2* (0.160 mM) or a control morpholino (0.160 mM). **(a–d)** Quantification of the comet run metrics extracted from kymograms show no difference in microtubule growth kinetics. **(a)** Average comet distance for AMO 0.160 mM (*p* = 0.8230) compared to AMO control condition. **(b)** Average comet duration for AMO 0.160 mM (*p* = 0.7340) compared to AMO control condition. **(c)** Average comet velocity for AMO 0.160 mM (*p* = 0.4605) compared to AMO control condition. **(d)** Average comet density for AMO 0.160 mM (*p* = 0.3741). Unpaired *t*-test **(a)** or Mann-Whitney test **(b,c,d)**; *N* = 3 experiments; *n* = 20–37 zebrafish embryos with each data point representing an individual zebrafish embryo. **(e)** Representative image (composite z-stack projection) of EB3-GFP in AMO control zebrafish embryo. Red box: representative area for the comet analysis in a distal segment in the CaP. **(f)** Representative kymograms represent microtubule growth extracted through time-lapse imaging (500 ms/5 min) of EB3-GFP comets in single CaP motor neurons distal arbors at 48 hpf. Each pixel on the *Y*-axis represents one timepoint image (time) projected against the neurite length (distance) on the *X*-axis. AMO, morpholino; hpf, hours post fertilization; CaP, caudal primary; scale bar, 25 μm; t, time (5 min); d, distance (average: 17 μm segments).

Microtubule growth rates are known to be affected by the amount of soluble, free tubulin (Stepanova et al., 2003). Since TUBA4A is highly expressed in neurons, knockdown of this specific α-tubulin isotype could alter microtubule assembly and thus be responsible for the observed phenotypes. We therefore investigated changes in microtubule polymerization by performing an EB3 comet assay. Indeed, EB3 is a plus-end tracking protein (+TIP) known to bind the plus-end of growing microtubules in order to regulate their dynamics (Stepanova et al., 2003). These assembly events, called ‘comets’, can be quantified by imaging the association of an EB3 fusion protein with the growing microtubule via time-lapse imaging. We performed a live, *in vivo* comet assay in single CaP motor neurons of 48 hpf zebrafish embryos injected with pUAS-EB3-GFP, in the mnx1:GAL4 background (Figure 6e: composite z-stack projection of one CaP motor neuron), as performed previously (Bercier et al., 2019). We quantified the comet metrics (i.e., distance, duration and velocity) on kymograms extracted from time-lapse imaging (Figure 6f) in *tuba8l2* AMO and control AMO injected embryos, but did not observe any significant changes in the kinetics of polymerization events (Figures 6a–c; respectively, *p* = 0.8230, unpaired *t*-test *p* = 0.7340, *p* = 0.4605, Mann-Whitney test). In addition, reduction of tuba8l2 did not affect the number of events, as quantified by the comet density (Figure 6d; *p* = 0.3741, Mann-Whitney test). Overall, a reduction of tuba8l2, without changes in total α-tubulin (Figures 2d, e), did not affect microtubule polymerization rates or kinetics in 48 hpf zebrafish motor neurons.

### 3.5 Changes in post-translational modifications of tubulin are induced by knockdown of *tuba8l2*

As we observed no significant changes in microtubule growth after knockdown of the *TUBA4A* zebrafish orthologue *tuba8l2*, we decided to look further into microtubule PTMs. Indeed, the isotype composition of microtubules can not only influence their stability, but also their PTMs which often decorate the C-terminal tail of tubulins regulating a range of functions (Janke and Magiera, 2020). More specifically, we investigated acetylated α-tubulin, detyrosinated α-tubulin, and polyglutamylated tubulin levels because all three have been shown to play a role in microtubule functionality (Bodakuntla et al., 2021; Cappelletti et al., 2021; Sanyal et al., 2021).

**FIGURE 7:**
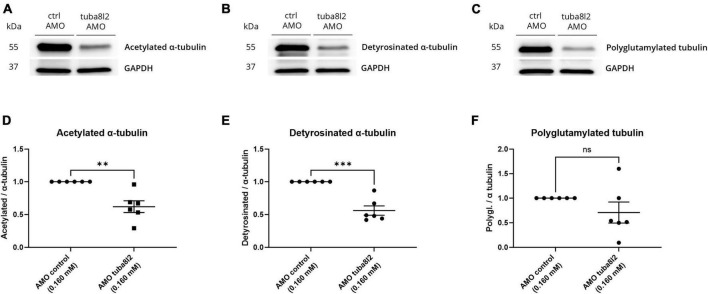
Specific *tuba8l2* knockdown in zebrafish induces changes in post-translational modifications of tubulin. **(a–f)** Western blot was performed on whole fish lysate from 48 hpf embryos injected with ATG morpholino against *tuba8l2* (0.160 mM) vs. control morpholino (0.160 mM). *N* = 6 experiments; *n* = 10–30 zebrafish per group per experiment. **(a–c)** Representative western blot for acetylated α-tubulin **(a)**, detyrosinated α-tubulin **(b)** and polyglutamylated tubulin **(c)**. **(d–f)** Quantification of acetylated α-tubulin **(d)** (*p* = 0017), detyrosinated α-tubulin **(e)** (*p* < 0.0001) and polyglutamylated tubulin **(f)** (*p* = 0.2014) protein levels relative to α-tubulin after normalization to GAPDH for the different injection conditions. Data represent mean ± SEM. Unpaired *t*-test: ** *p* < 0.01, *** *p* < 0.001. AMO, morpholino; hpf, hours post fertilization; ctrl, control.

First, we performed western blotting on total lysate from 48 hpf zebrafish embryos injected with *tuba8l2* AMO compared to control AMO (Figures 7a–c). We found that both acetylated as well as detyrosinated α-tubulin levels were significantly decreased upon knockdown of *tuba8l2* (Figures 7d, e; respectively, *p* = 0017, *p* = 0.0001 unpaired *t-*test). For polyglutamylated tubulin on the other hand, there was more variation although a general trend toward decreased levels could be observed (Figure 7f, *p* = 0.2014 unpaired *t-*test). These results suggest that a reduction in tuba8l2 affects microtubule PTMs, which are known to be involved in neurodegeneration, and therefore potentially affect the functions they regulate, such as interactions with molecular motors for axonal transport, protection from disassembly and/or from mechanical aging (Janke and Magiera, 2020; Moutin et al., 2021).

## 4 Discussion

In this study, we showed that a reduction of TUBA4A protein expression, as observed in *post-mortem* tissue of sporadic ALS patients, leads to an ALS-related phenotype in embryonic zebrafish via knockdown of the zebrafish orthologue of *TUBA4A* (*tuba8l2)*. Moreover, we observed a decreased axonal length of spinal motor neurons and a behavioral phenotype, which were dose-dependent and rescued by the addition of wild-type human *TUBA4A* mRNA, demonstrating the specificity of our approach and conservation of the orthologue. We hereby investigated the mechanism by which the reduction of tuba8l2 could induce these phenotypes and found no change in microtubule polymerization while we did find alterations in post-translational modifications of tubulin, known to be involved in neurodegeneration. These results extend the importance of *TUBA4A*, a familial ALS disease gene, to sporadic ALS cases, suggesting that alterations in its expression may indeed be a contributing factor in ALS pathophysiology.

Mutations in various tubulin isotypes are associated with disease, supporting a functional specification of alternative isotypes (Breuss et al., 2017). Interestingly, *TUBA4A* is the isotype with the highest expression in the human motor cortex after birth (Leandro-García et al., 2010; Sferra et al., 2020) and its expression in the brain dramatically increases with age. This could explain why *TUBA4A* dysregulation may contribute to adult-onset neurodegenerative disease, contrary to mutations in other tubulin isotypes involved in neurodevelopmental disorders (Tischfield et al., 2011; Chakraborti et al., 2016; Castellanos-Montiel et al., 2020). Previously, C-terminal mutations in *TUBA4A* were shown to be associated with classical spinal onset ALS, and to be associated in some cases with FTD-like symptoms (Smith et al., 2014; Perrone et al., 2017). The C-terminal part of TUBA4A is important for its interaction with β-tubulin and microtubule-associated proteins (MAPs), which regulate microtubule stability (Wall et al., 2016). C-terminally mutated TUBA4A proteins were shown to be ineffective at forming tubulin dimers *in vitro*, and displayed a decreased incorporation into protofibrils, inhibiting microtubule network stability (Smith et al., 2014). These mutations, would therefore represent a toxic gain-of-function due to the production of mutant protein products. On the other hand, N-terminal *TUBA4A* mutations were identified in patients presenting with FTD, possibly with extrapyramidal symptoms (Perrone et al., 2017; Mol et al., 2021). We and others have shown that these mutations led to reduced TUBA4A levels in central nervous system tissues, suggesting a loss-of-function mechanism (Mol et al., 2021; Okada et al., 2021; Van Schoor et al., 2022).

Interestingly, a downregulation of TUBA4A protein expression was also reported in the brain of sporadic ALS patients (Helferich et al., 2018), which we confirmed in this study via western blotting of motor cortex tissue. In addition, we detected a trend toward a decreased TUBA4A expression in the spinal cord of sporadic ALS patients, though the high variability between samples prevented statistical significance. Furthermore, qualitative IHC analysis indicated no differences in the cellular localization of TUBA4A between ALS and control cases, and no TUBA4A aggregates were detected, in line with a previous study (Smith et al., 2014). Altogether, these data suggest that alterations in expression of *TUBA4A* are of importance in sporadic ALS pathogenesis.

Downregulation of TUBA4A can occur through the miR-1825/*TBCB*/*TUBA4A* pathway, previously reported to be dysregulated in sporadic and familial ALS. Indeed, it was demonstrated that miR-1825 downregulation in ALS patient tissue led to increased tubulin-folding cofactor B (TBCB) levels, which led to the sequestration of TUBA4A and induced a decrease in its expression (Helferich et al., 2018). This gives an indication of the possible upstream events leading to decreased TUBA4A levels in ALS, aside from N-terminal *TUBA4A* mutations (Van Schoor et al., 2022). Importantly, we here demonstrate the causality of TUBA4A downregulation by showing that knockdown of the *TUBA4A* orthologue *tuba8l2* in zebrafish embryos led to ALS-associated abnormalities, namely spinal axonopathy and behavioral deficits. These phenotypes were previously also shown to be triggered by pathological protein products such as mutant SOD1 (Van Hoecke et al., 2012) and mutant TDP-43 (Laird et al., 2010), as well as by the pathological hallmarks of patients with *C9orf72* mutations, i.e., dipeptide repeat proteins (DPRs) and sense and anti-sense repeat RNA (Swinnen et al., 2018), among others. Importantly, the severity of the observed phenotypes was dose-dependent, did not affect total α-tubulin levels and could be rescued by co-expression of human TUBA4A, which alone caused a reduction of endogenous tuba8l2 (Online resource; Supplementary Figure 4). This suggests that the TUBA4A level is specifically important for neuronal health and that its reduction can contribute to ALS pathobiology.

Since a reduction of TUBA4A might affect cytoskeletal integrity in neurons, we investigated microtubule polymerization in our zebrafish model. However, we did not observe changes in the number of polymerization events or the kinetics at which they occur. We then assessed the levels of known PTMs in whole fish lysate and found decreased levels of acetylated α-tubulin, detyrosinated α-tubulin, and trends indicating decreased polyglutamylated tubulin. Indeed, aside from the composition of specific tubulin isotypes, microtubule specification also occurs through tubulin post-translational modifications (PTMs). Specifically, neuronal microtubules mainly undergo detyrosination of the C-terminal tyrosine, acetylation at K40 and polyglutamylation (Moutin et al., 2021). These PTMs are fine-tuned, unevenly distributed and are known to accumulate as neurons differentiate and mature (Sferra et al., 2020). Importantly, out of nine α-tubulins isotypes, only TUBA8 and TUBA4A lack the C-terminal tyrosine residue which undergoes detyrosination (Janke and Magiera, 2020; Sanyal et al., 2021). As a result, the integration of TUBA4A in microtubules mimics enzymatically detyrosinated α-tubulin, linked with increased stability (Janke and Magiera, 2020), and is enriched in the axonal microtubules of neurons (Boiarska and Passarella, 2021; Moutin et al., 2021; Sanyal et al., 2021). A reduction in TUBA4A could therefore lead to decreased levels in detyrosinated α-tubulin, in line with what we have observed. Furthermore, acetylation is present along the entire axonal microtubule cytoskeleton, while tyrosination is predominantly present at the growing, actively polymerizing ends of microtubules. Axonal microtubules are thus presumed to be long-lived, having highly acetylated and detyrosinated tubulins (Janke and Magiera, 2020). According to our findings in the zebrafish model, the reduction of *TUBA4A* by knockdown of orthologue *tuba8l2* results in decreased levels of both acetylated and detyrosinated α-tubulin. While we could not achieve statistical significance for the polyglutamylated tubulin levels, our results do suggest a decrease which could have an additional negative impact on microtubule functionality. Indeed, polyglutamylation does not only modulate the binding of MAPs and molecular motors, it is also shown to be necessary in modulating synaptic transmission-related transport. Furthermore, alterations in this PTM were previously associated with neurodegeneration (Magiera et al., 2018; Moutin et al., 2021; Hausrat et al., 2022).

In ALS, the dying-back hypothesis implies that abnormalities in axon connectivity and synaptic function long precede somatic cell death (Fischer et al., 2004; Nijssen et al., 2017). Defects in cytoskeleton integrity and microtubule-dependent transport mechanisms can thus result in axonal trafficking disruption and dysfunctional neuromuscular junctions (Castellanos-Montiel et al., 2020; Sanyal et al., 2021; Stoklund Dittlau et al., 2021). Due to the physical length of motor neuron axons, cortical and spinal motor neurons are thought to be particularly vulnerable to this dying-back mechanism (Clark et al., 2016). Apart from *TUBA4A*, other genes involved in cytoskeleton integrity have also been linked with ALS, namely *DCTN1*, *KIF5A*, *PRPH*, *NF*-*H*, *PFN1* and *SPAST* (Chakraborti et al., 2016; Guo et al., 2020). This stresses the causative relation between cytoskeletal defects and neurodegeneration in the context of ALS. Therefore, our results are consistent with the hypothesis that a downregulation of TUBA4A expression leads to aberrant cytoskeletal function, explaining the observed axonal phenotype and motor behavior in zebrafish.

In conclusion, we showed an ALS-related axonopathy and behavioral phenotype in zebrafish embryos following downregulation of the zebrafish orthologue for *TUBA4A* via knockdown of *tuba8l2*. While we did observe changes in tubulin PTMs, we did not observe changes in microtubule polymerization. This could be due to the fact that expression of total α-tubulin was not affected by the reduction of tuba8l2 (Wethekam and Moore, 2022). Overall, these data support that, apart from ALS cases bearing a *TUBA4A* mutation, dysregulated TUBA4A expression plays an important role in sporadic ALS disease pathogenesis, and stresses the importance of microtubule dysfunction in ALS.

### 4.1 Limitations of this study

In this study, we assessed axonal and behavioral phenotypes in embryonic zebrafish stages (until 48 hpf). An important limitation of the use of zebrafish to model a motor neuron disorder such as ALS is the absence of corticospinal upper motor neurons projecting to the spinal cord in zebrafish. The use of embryonic zebrafish also has its limitations to model an adult onset neurodegenerative disease. However, zebrafish experiments offer an advantage over standard cell culture models as it gives the possibility to study effects on motor behavior in addition to axonal pathology, which is not possible *in vitro*. Moreover, the zebrafish orthologue *tuba8l2* shows 94% conservation compared to human *TUBA4A* at the protein level, and we showed that TUBA4A can compensate for the loss of tuba8l2 in our rescue experiments. This suggests functional conservation between the two orthologues, with both genes able to regulate motor axon morphology and motor behavior. Although the use of morpholino-based knockdown in zebrafish can be an important avenue to explore emerging pathogenic pathways in neurodegenerative disorders, like any model, it has its limitations. Thus, research including other *in vivo* and patient-relevant *in vitro* models is needed to further unravel the downstream consequences of TUBA4A downregulation, and how this contributes to ALS-related neurodegeneration.

## Data availability statement

The raw data supporting the conclusions of this article will be made available by the authors, without undue reservation.

## Ethics statement

Brain and spinal cord tissues were collected in accordance with the applicable laws in Belgium (UZ Leuven) and Germany (Ulm). The recruitment protocols for collecting the brains were approved by the ethical committees of the University of Ulm (Germany) and of UZ Leuven (Belgium) and consent was obtained.

## Author contributions

EVS: Conceptualization, Data curation, Formal Analysis, Investigation, Methodology, Visualization, Writing–original draft, Writing–review and editing. DS: Formal Analysis, Investigation, Methodology, Validation, Visualization, Writing–original draft, Writing–review and editing. EB: Formal Analysis, Investigation, Methodology, Visualization, Writing–review and editing. JW: Resources, Writing–review and editing. ACL: Resources, Writing–review and editing. PVD: Conceptualization, Funding acquisition, Resources, Writing–review and editing. DRT: Conceptualization, Funding acquisition, Resources, Supervision, Writing–review and editing. VB: Conceptualization, Formal Analysis, Methodology, Supervision, Visualization, Writing–original draft, Writing–review and editing. LVDB: Conceptualization, Funding acquisition, Supervision, Writing–review and editing.
