# Supplementary material for: TUBA4A downregulation as observed in ALS post-mortem motor cortex causes ALS-related abnormalities in zebrafish
Source: Front Cell Neurosci. 2024 Feb 21;18:1340240. doi: 10.3389/fncel.2024.1340240 (PMC10921936; doi:10.3389/fncel.2024.1340240)
Supplement: Supplementary file 1 [file Data_Sheet_1.PDF]

Supplementary Material:

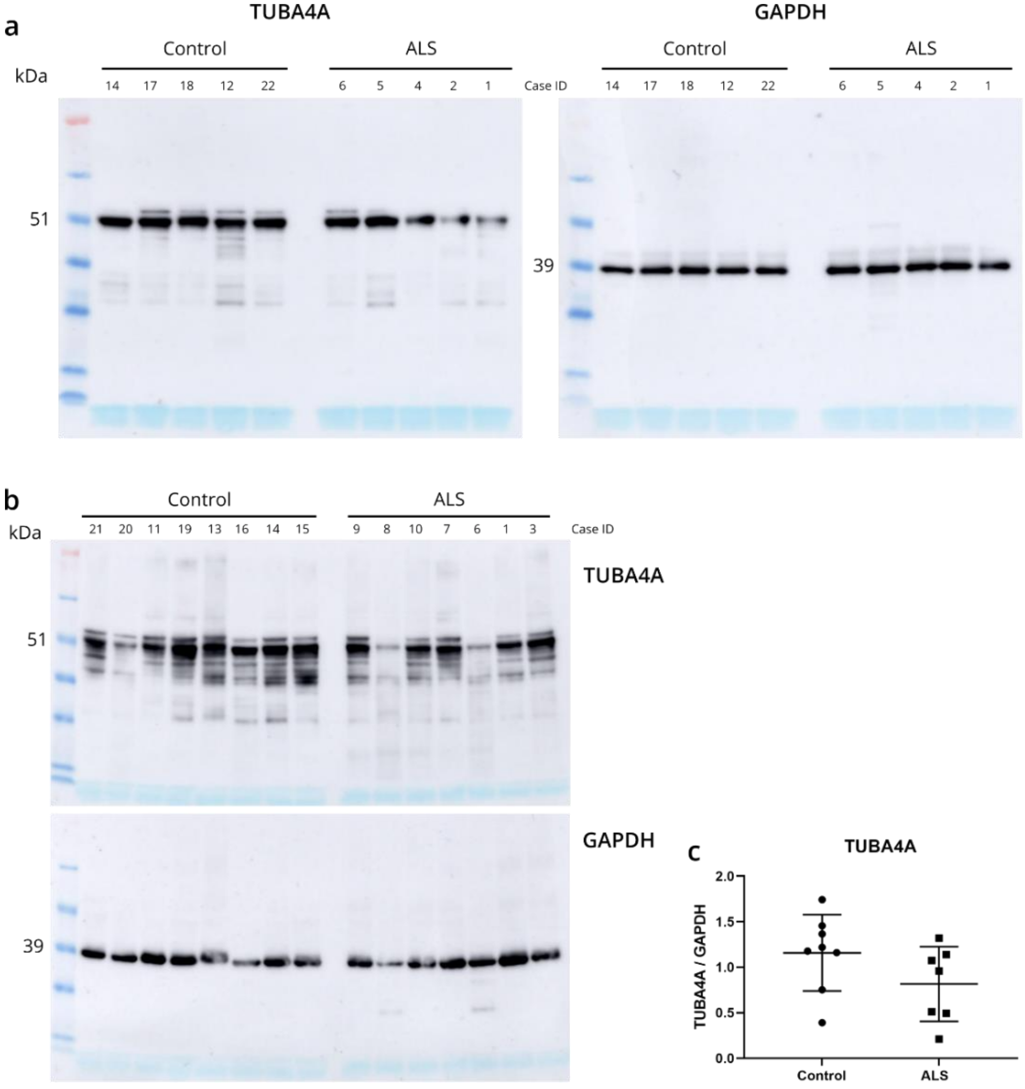

Supplementary Figure 1. ALS post-mortem motor cortex and spinal cord full western blots for TUBA4A. (a,b) Full western blots using antibodies against TUBA4A and GAPDH on SDS-soluble lysates extracted from the motor cortex (a) and spinal cord (b) of control (n = 5 and n = 8 resp.) and ALS (n = 5 and n = 7 resp.) cases. **(c)** Quantification of the expression of TUBA4A as a ratio to GAPDH in the spinal cord. p = 0.1349; unpaired t-test.

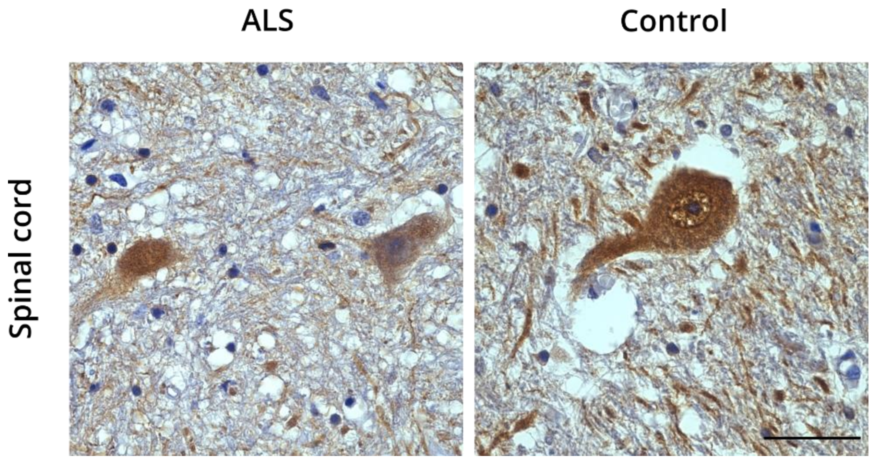

Supplementary Figure 2. Immunohistochemical analysis of TUBA4A in ALS and control *post-mortem* cases. Immunohistochemical stainings of spinal cord of representative ALS and control cases with an antibody against TUBA4A. Scale bar represents 50 µm.

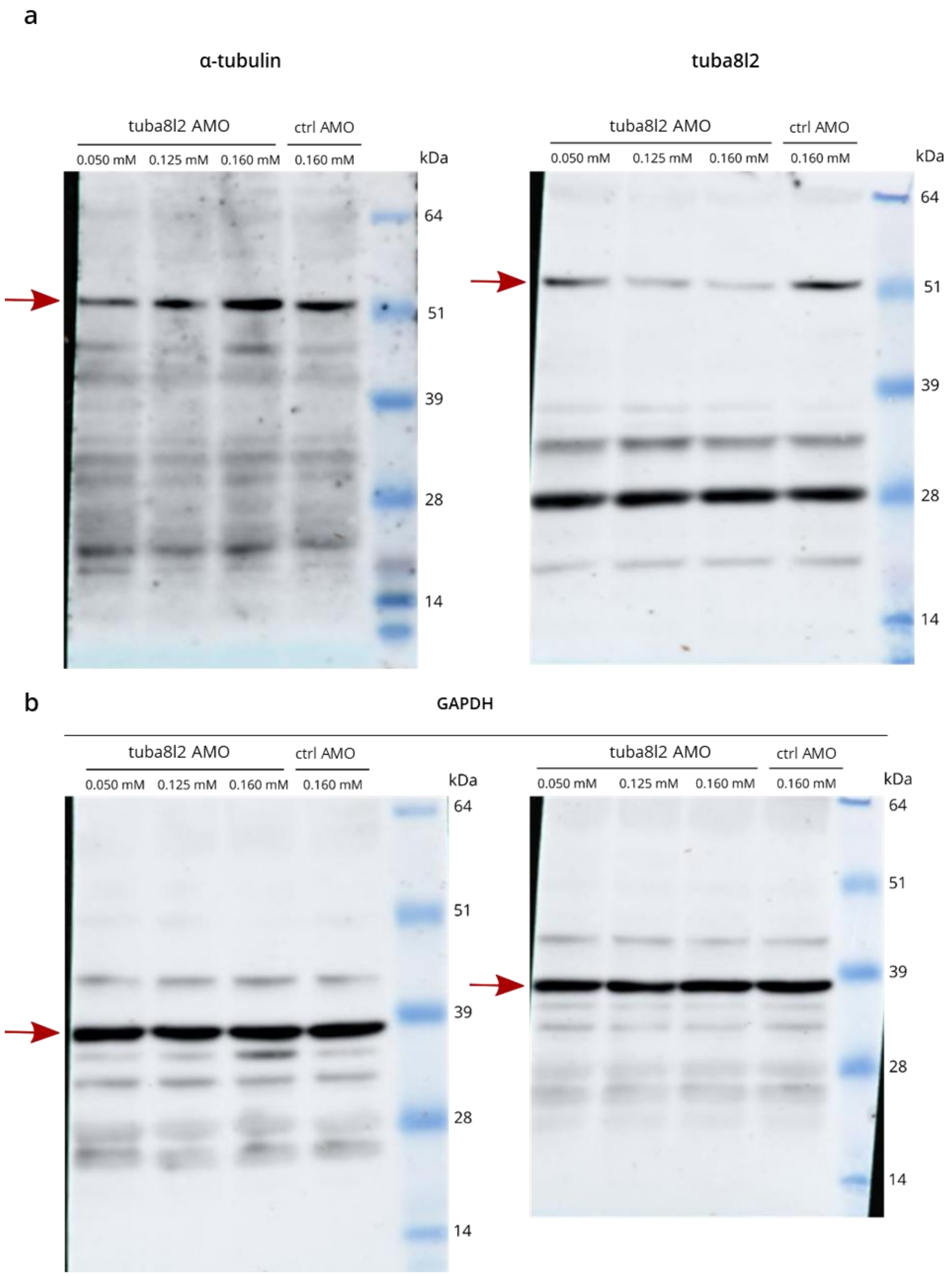

Supplementary Figure 3. Full western blots showing the specific tuba8l2 knockdown in zebrafish. Western blot was performed at 48 hpf after injection of different doses of ATG morpholino against tuba8l2 (0.160 mM, 0.125 mM and 0.050 mM) as well as the injection of a control morpholino (0.160 mM). **(a)** Arrow indicates the correct band for α-tubulin and tuba8l2. **(b)** Arrow indicates the correct band at 37 kDa for the corresponding GAPDH loading control.

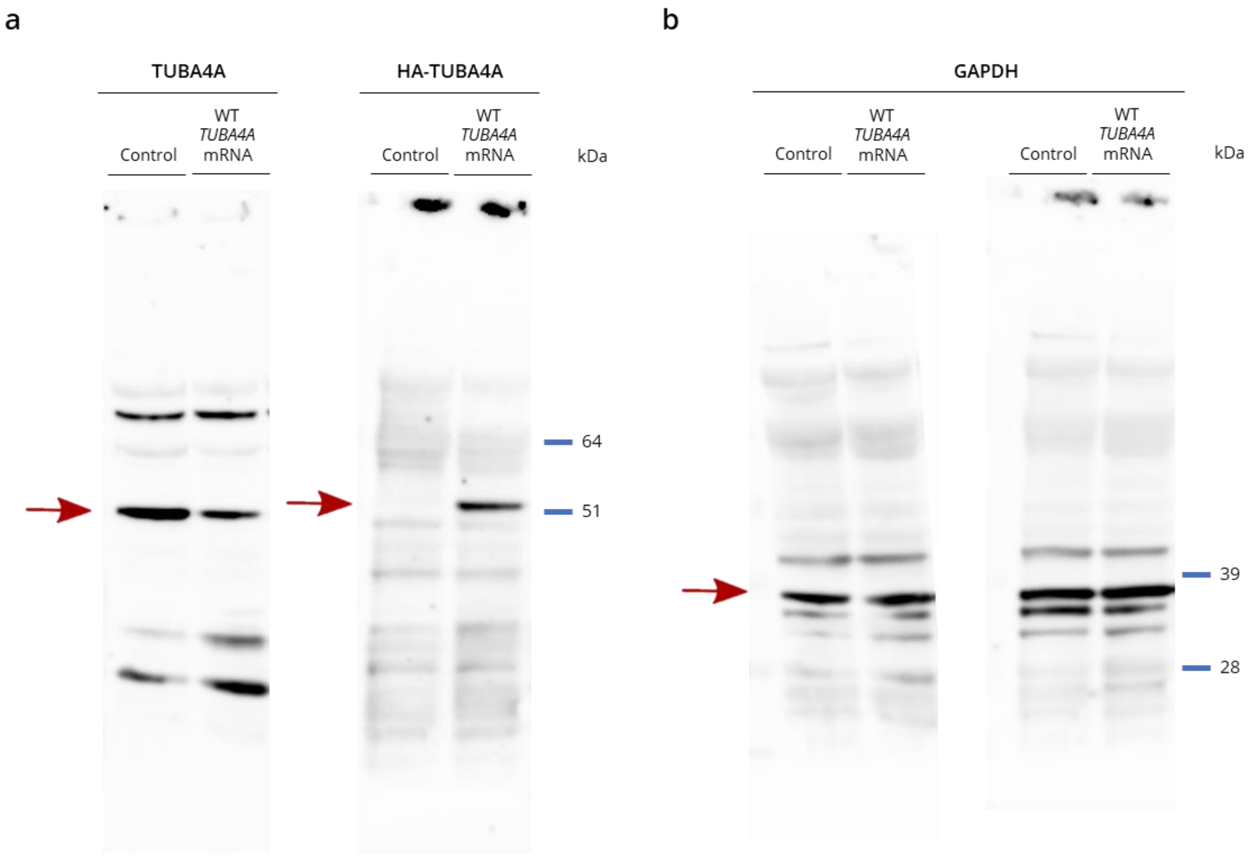

Supplementary Figure 4. Confirmation of expression of HA-tagged wild-type TUBA4A protein after micro-injection of mRNA in zebrafish. Western blot performed at 48 hpf after injection of HA-tagged wild-type *TUBA4A* mRNA shows **(a)** a down regulation of endogenous TUBA4A expression (left) with the expression of an HA-TUBA4A protein product using an antibody against HA (cat no. 3724, Cell Signaling, Danvers, MA, USA, 1:1000) (right). This was absent in the non-injected control condition. Arrow indicates the correct band for TUBA4A (left) and HA-TUBA4A (right). **(b)** Arrow indicates the correct band at 37 kDa for the corresponding GAPDH loading control.

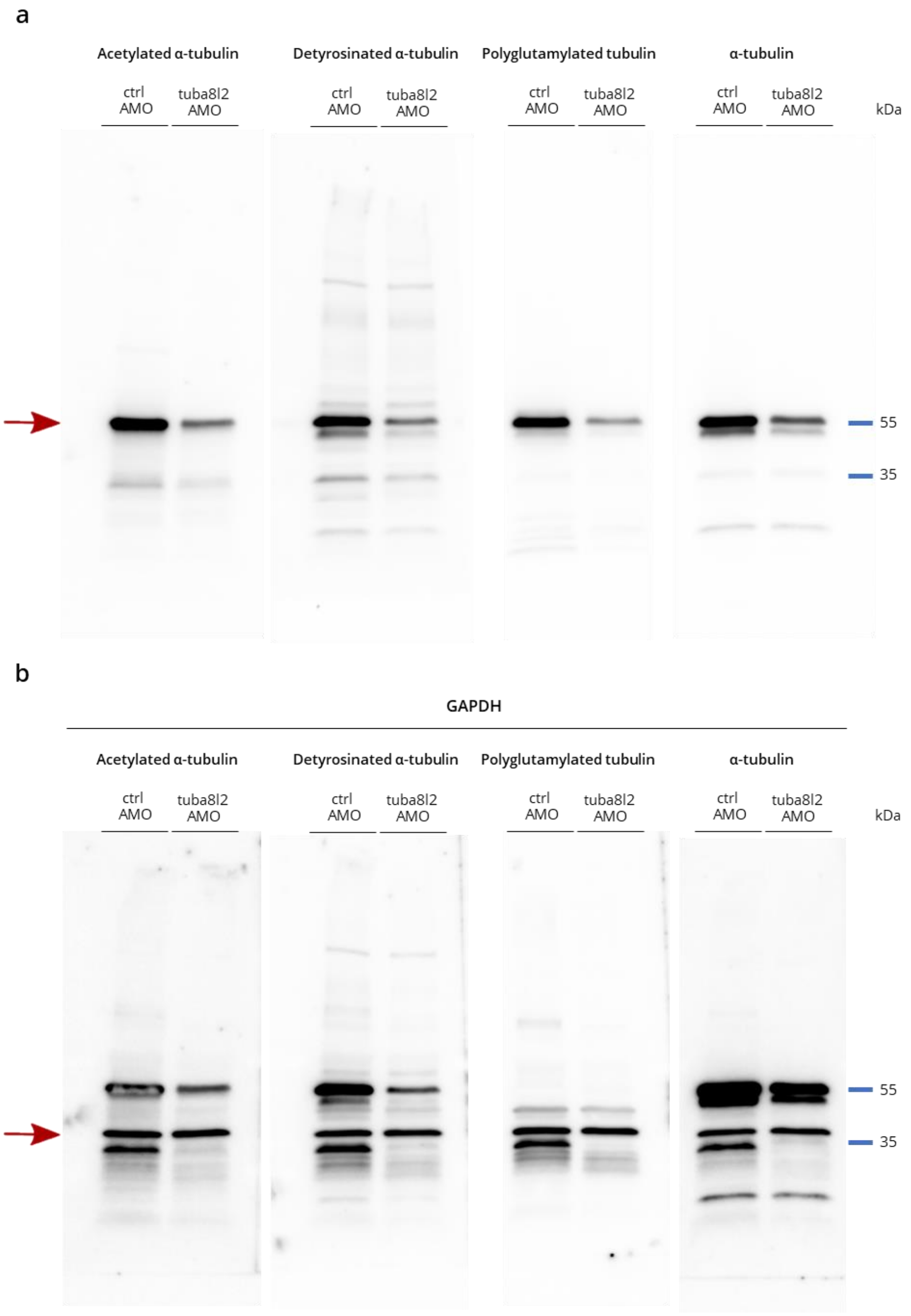

Supplementary Figure 5. Full western blots showing the post-translational modifications for tuba8l2 knockdown in zebrafish. Western blot was performed at 48 hpf after injection of ATG morpholino against tuba8l2 (0.160 mM) as well as the injection of a control morpholino (0.160 mM). **(a)** Arrow indicates the correct band for acetylated α-tubulin, detyrosinated α-tubulin, polyglutamylated tubulin and α-tubulin. **(b)** Arrow indicates the correct band at 37 kDa for the corresponding GAPDH loading control.

Supplementary Table 1. List of human cases used in the study. The table provides information regarding age (= age at death), sex, diagnosis, AβMTL phase, Braak NFT stage, disease duration (months), PMI (hours) and application (1 = western blot motor cortex; 2 = western blot spinal cord; 3 = immunohistochemistry). Abbreviations: f = female; m = male; ALS = amyotrophic lateral sclerosis; control = non-neurodegenerative disease control; A = aneurysm; AGD = argyrophilic grain disease; ARTAG = aging-related tau astrogliopathy; CM = carcinoma metastasis; GB = Guillian-Barre syndrome; I = infarction; MI = microinfarction; n.a. = not applicable; PART = primary age-related tauopathy; SVD = small vessel disease; SVE = subcortical vascular encephalopathy; PMI = *post-mortem* interval.

Case n°	Age	Sex	Diagnosis	Aβ MTL phase	Braak NFT stage	Disease duration	PMI (hours)	Application
1	58	f	ALS, PART	0	2	18 months	24	(1,2,3)
2	51	m	ALS	0	1	8 months	24	(1)
3	49	m	ALS, PART	0	1	45 months	24	(2)
4	46	m	ALS	0	1	40 months	24	(1)
5	62	m	ALS, MI, I, ARTAG, PART, AGD	0	1	154 months	12	(1)
6	53	m	ALS	0	1	92 months	24	(1,2,3)
7	74	m	ALS	1	1	47 months	24	(2)
8	50	f	ALS	0	1	18 months	24	(2)
9	54	m	ALS	0	1	88 months	6	(2)
10	68	m	ALS, SVD	2	2	36 months	144	(2)
11	46	m	Control	0	1	n.a.	29	(2)
12	74	m	Control, CM, I, MI	0	0	n.a.	72	(1,3)
13	45	m	Control	0	0	n.a.	24	(2)
14	61	m	Control, SVD	0	0	n.a.	48	(1,2,3)
15	55	m	Control, A, I	0	0	n.a.	96	(2)
16	74	f	Control, AGD, SVE	0	1	n.a.	24	(2)
17	35	m	Control, Limbic encefalopathy	0	0	n.a.	72	(1)
18	54	m	Control, GB	0	1	n.a.	24	(1)
19	63	f	Control, MI	0	1	n.a.	96	(2)
20	64	m	Control	0	0	n.a.	96	(2)
21	35	m	Control	0	1	n.a.	48	(2)
22	64	m	Control	0	1	n.a.	24	(1)

Supplementary Table 2. List of antibodies used in the study. The table summarizes information about host, clonality, supplier and catalog number of the primary antibodies used for immunohistochemistry (IHC), immunofluorescence (IF) and western blot (WB). Dilutions are given.

Primary antibody	Host	Clonality	Supplier	Catalogue number	Dilution IHC	Dilution IF	Dilution WB
TUBA4A (C-term)	Rabbit	Polyclonal	Abgent	AP13535b	1:100	-	1:8000
alpha-tubulin	Mouse	Monoclonal	Sigma	T6199	-	-	1:2000
anti-GAPDH (clone 6C5)	Mouse	Monoclonal	ThermoFisher	AM4300	-	-	1:10 000
anti-β-Amyloid (clone 4G8)	Mouse	Monoclonal	BioLegend	SIG-39220	1:5000	-	-
anti-pTDP43 (S409/410-2)	Rabbit	Polyclonal	Cosmo Bio	TIP-PTD-P02	1:5000	-	-
anti-pTau (S202/T205) (clone AT8)	Mouse	Monoclonal	ThermoFisher	MN1020	1:1000	-	-
SV2	Mouse	Monoclonal	DSHB	AB2315387	-	1:200	-
Anti-Acetylated tubulin	Mouse	Monoclonal	Sigma	T7451	-	-	1:5000
Anti-Detyrosinated tubulin	Rabbit	Polyclonal	Sigma	AB3201	-	-	1:500
Anti-polyglutamate (IN105)	Rabbit	Polyclonal	Adipogen	AG-25B-0030-C050	-	-	1:20 000
